# Effect of clover sprouts protein hydrolysates as an egg substitute on physicochemical and sensory properties of mayonnaise

**DOI:** 10.1002/fsn3.2665

**Published:** 2021-12-06

**Authors:** Dina Mirsadeghi Darabi, Peiman Ariaii, Reza Safari, Mohammad Ahmadi

**Affiliations:** ^1^ Department of Food Science and Technology, Ayatolla Amoli Branch Islamic Azad University Amol Iran; ^2^ Agricultural Research Education and Extension Organization Caspian Sea Ecology Research Institute Iranian Fisheries Science Research Institute Sari Iran

**Keywords:** amino acid, commercial enzyme, mayonnaise, physicochemical properties, protease

## Abstract

Mayonnaise is a semi‐solid oil‐in‐water emulsion that in addition to eggs other stabilizers and thickeners are used as emulsifiers for better stability. Although eggs are an important ingredient in the production of mayonnaise, the health problems associated with the use of eggs is increasing due to their high cholesterol content. The aim of this study was to evaluate the feasibility of clover sprout protein hydrolysates (CSPH) to replace eggs for the production mayonnaise. First, CSPH was produced using alcalase and flavourzyme enzyme, and in order to find the best enzyme, the degree of hydrolysis (DH) and protein recovery (PR) were determined. Then four mayonnaise treatments included, T1: control (egg 9%), T2: egg 6%+ CSPH 3%, T3: egg 3%+ CSPH 6%, T4: egg 0%+ CSPH 9% was prepared and the stability, viscosity, physicochemical, textural, and sensory properties of mayonnaise was investigated. The samples containing CSPH showed that CSPH had high essential amino acids, CSPH from alcalase enzyme had higher amounts of protein, DH, PR, and increasing hydrolysis time had a positive effect on these parameters (*p* < .05); therefore, CSPH from alcalase enzyme was used for the production mayonnaise. The stability, viscosity, firmness, adhesion of texture, and pH increased with increasing CSPH, while the brightness, acidity, and sensory score of the samples decreased (*p* < .05). In general, T3 had an acceptable quality in terms of the studied characteristics, but sensory score in T4 could not be confirmed. Hence, by replacing eggs with CSPH up to 6%, mayonnaise with appropriate physicochemical and sensory properties can be produced. Therefore, the formulation egg 3%+ CSPH 6% is an appropriate choice to produce mayonnaise for consumers who are on a restricted diet to eat foods containing eggs.

## Graphical abstract

Mayonnaise is an oil‐in‐water emulsion that in addition to eggs other stabilizers and thickeners are used as emulsifiers for better stability. Although eggs are an important ingredient in the production of mayonnaise, the health problems associated with the use of eggs is increasing due to their high cholesterol content. The aim of this study was to evaluate the feasibility of clover sprout protein to replace eggs for the production mayonnaise. The results indicate that using clover protein up to 6% can satisfactorily remove most of the mayonnaise egg and produce low‐egg mayonnaise, thus producing a functional product and preventing the side effects of egg yolk consumption.

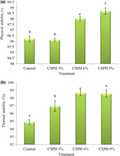

## 1 INTRODUCTION

Mayonnaise is a semisolid oil‐in‐water emulsion that contains 70%–80% fat and is prepared conventionally by mixing eggs, vinegar, oil, and spices, especially mustard (Katsaros et al., 2020). Eggs are an important ingredient in the production of mayonnaise due to their nutritional and functional roles, including emulsification, foaming, and stability, and constitute the dominant structure in the continuous phase (Ali and El Said, 2020). However, the health problems associated with the use of eggs is increasing due to their high cholesterol content (Karshenas et al., 2018; Pradhananga and Adhikari, 2015). In general, there is a close relationship between high cholesterol and the incidence of cardiovascular disease. Studies showing the link between egg consumption and cardiovascular disease have led consumers to consider mayonnaise as a unhealthy food (Karshenas et al., 2018).

The use of plant sources as a substitute for animal proteins has a long history and dates back to the 13th century. During that time, their use was especially common in infant food formulation due to the presence of high amount of protein, minerals, and vitamins in these plant sources. So far, some plant proteins (e.g., gluten, wheat protein, clover germ protein, and isolate and concentrate of whey protein) with suitable functional properties have been evaluated as an alternative to eggs alone or in combination with gums in oil‐in‐water emulsions. (Alu'datt et al., 2017).

Persian clover (*Trifolium resupinatum* L.) is an annual forage plant that grows in semiarid conditions of the Mediterranean region. Clover sprout protein, which is rich in water‐soluble and salt‐soluble proteins, has desirable functional properties such as emulsifying activity, high water retention ability, foaming, and high solubility that makes it a useful compound for use in food products (Ates, 2011; Khatami et al., 2016). Clover sprouts are also rich in amino acids, especially essential amino acids such as lysine, methionine, and threonine, which are rare in many cereal grains; therefore, it is considered as one of the most important and valuable sources of plant proteins. Due to the fact that clover sprouts, with their high nutritional value, have beneficial effects on human health and are a cheap source of protein, the protein in it can be considered as one of the best sources of protein. Plants are known to be extracted or hydrolyzed for human consumption to be used in food formulations (Ates, 2011; Yunhu et al., 2006). Hydrolysis of proteins is actually breaking them down into smaller peptides and free amino acids. Enzymatic hydrolysis is a complex process and many preliminary studies are necessary to understand and create an effective enzymatic process. The enzyme must be able to hydrolyze specific peptide bonds in the protein chain to produce antioxidant peptides. Hydrolysis‐independent variables have a direct effect on enzyme activity and consequently on the antioxidant properties of the final peptides. Antioxidant peptides are economically preferred because they are also effective at low concentrations. The most important enzymes used in various studies include alcalase (which has endoprotease activity in alkaline conditions) and flavourzyme (a mixture of endopeptidase and exopeptidase) (Hamzeh et al., 2019; Nemati et al., 2012; Ngo et al., 2012; Varedesara et al., 2021; Yaghoubzadeh et al., 2020).

Little research has been carried out on the production of low cholesterol products, especially mayonnaise, and there are no reports on the use of hydrolyzed clover sprout protein as an egg substitute in mayonnaise formulation in Iran and the world, Therefore, the aim of this study was to evaluate the feasibility of clover sprout protein to replace eggs for the production of low‐cholesterol mayonnaise with high nutritional value and low cost of production. For this to purpose, protein produced by alcalase and flavourzyme enzymes from clover sprouts was hydrolyzed and its effect as an egg substitute on the physicochemical and sensory properties of mayonnaise was investigated.

## 2 MATERIALS AND METHODS

### 2.1 Preparation of raw materials

Clover sprouts (Ravak brand) were purchased under from the Arian Store. Alcalase (extracted from *Bacillus* *licheniformis*) and flavourzyme (extracted from *Aspergillus* *oryzae*) were purchased from Novazim and stored at 4°C until the further use. All the chemicals used in the experiments were purchased by the Merck Company and were laboratory grade.

### 2.2 Production of clover sprout protein isolates

To produce hydrolyzed protein, clover sprouts were ground using a laboratory hammer mill (Azhand pajouhan) after degreasing with *n*‐hexane at dry room temperature (25 ± 1°C). Clover sprouts flour was stored at 4°C until use after sieving with a 70‐mesh screen (0.0083 inch) (Zhu et al., 2006). The flour was stirred in 1 NaCl mol/L solution (1:8 w/v) at room temperature (25 ± 1°C) for 30 min and then the pH was adjusted to 9.5. After stirring for 30 min, the suspension was centrifuged at 800 rpm for 20 min at 4°C. The supernatant was adjusted to pH 4 using 1 mol/L HCl to precipitate the proteins and centrifuged again at 800 rpm for 20 min at 4°C. The precipitates were washed several times with distilled water and adjusted to pH 7 in distilled water containing NaOH. Scattered particles were dried using a freeze dryer (FDB‐550, Operon) (Zhu et al., 2006).

### 2.3 Isolation of protein isolated from clover sprouts

Fifty grams of the sample was poured into a 250‐ml Erlenmeyer flask and then 100 ml of distilled water was added in a ratio 2:1 to the Erlenmeyer flask and homogenized with a digital stirrer at 2000 *g* (DHS700) for 2 min. Then, the optimal pH for the activity of enzymes (8.5 for alcalase and 7 for flavourzyme) was achieved by adding 0.2 normal sodium hydroxide. The samples were placed in a mobile water bath at 50°C to produce a hydrolyzed protein at a constant speed of 200 rpm, then the enzyme (1% of the protein content of the clover sprout protein isolate) was added to it and at the end of the experiment (time 30 min), they were placed in a water bath for 15 min at 95°C to stop the enzymatic reaction. After cooling, the hydrolyzed proteins were centrifuged at a constant speed of 6700 *g* for 20 min, the floating liquid collected and the hydrolyzed protein was stored in the freezer, and then, it was pulverized using a freeze dryer. The degree of hydrolysis (DH) was calculated based on the amount of α‐amino acid in the sample protein (Chatterjee et al., 2015).

The amount of soluble protein in the supernatant was determined using the Biuret method. For this purpose, bovine serum albumin protein was used to draw the standard diagram. The measurement was performed at a wavelength of 540 nm using a spectrophotometer (DR 6000). The protein recovery (PR) rate was calculated using the following equation (Nemati et al., 2012):

Protein recovery = (amount of soluble protein in the hydrolyzed protein/amount of protein in the sample) × 100.

The hydrolyzed protein powder was completed for 24 h at 110°C using 6 N hydrochloric acid. Then, amino acid derivation was performed using phenylisothiocyanate (PITC). The amount of total amino acids was determined using HPLC model Smart line using a fluorescent detector (RF‐530, Knauer). The samples were then derivatized with *o*‐phthaldialdehyde (OPA) and analyzed using a C18 column (Knauer) at the flow rate of 1 ml/min with a fluorescence detector (Hamzeh et al., 2019).

### 2.4 Mayonnaise production

In this study, mayonnaise samples were produced in three steps. Mayonnaise was prepared with a formulation similar to one of the reputable domestic factories, except that the number of eggs was changed in different treatments and replaced with clover sprout protein (Table 1). To make mayonnaise samples, water, eggs, one‐third of vinegar, powdered ingredients including salt, sugar, mustard powder, and stabilizer were mixed together using a digital stirrer, and after complete mixing for 2 min, the oil was gradually added dropwise and then in a thin stream for 7 min. After forming the emulsion and creating a suitable texture, the remaining vinegar and water were added to the mixture until a completely homogeneous mixture was obtained. The final sample was transferred into glass containers and was kept in the refrigerator (LG; temperature of 4°C) until the experiments were performed. One kilogram of mayonnaise was prepared for each treatment. Treatment 1 (sample containing 9% eggs) was considered as a control sample. Due to the fact that fresh egg has about 70% moisture and 20% dry matter, the hydrolyzed clover sprout protein replaced the dry matter and was used as a water‐soluble suspension in each treatment. The moisture content of the samples was almost constant.

**TABLE 1:** Formulations and ingredients of mayonnaise (g/100 g)

Row	Compounds	T1	T2	T3	T4
1	Liquid oil	66	66	66	66
2	Vinegar (11%)	6	6	6	6
3	Eggs	9	6	3	0
4	Clover sprout protein	0	3	6	9
5	Water	12.35	12.35	12.35	12.35
6	Sugar	5	5	5	5
7	Salt	1.2	1.2	1.2	1.2
8	Mustard	0.3	0.3	0.3	0.3
9	Stabilizer	0.15	0.15	0.15	0.15

### 2.5 Determination of approximate compounds

Moisture, ash, crude protein, and fat content of mayonnaise were determined according to the AOAC (2005) method.

#### 2.5.1 Determination of pH

The pH of the samples was determined according to Institute of Standard and Industrial Research of Iran (ISIRI) No. 2454. A 5% solution of mayonnaise was prepared in a beaker (temperature 25°C) and the pH of the samples was measured using a pH meter (Lovibond) (ISIRI, 2014).

#### 2.5.2 Determination of acidity

To determine the acidity, ISIRI No. 2454 was used. Fifteen grams of the sample was diluted in 200 ml of distilled water and titrated with 0.1 N normal sodium hydroxide in the presence of phenolphthalein reagent until a light pink color appears. Acidity was calculated in terms of acetic acid according to the following formula (ISIRI, 2014):

Acidity (percentage) = (The volume of the consuming sodium hydroxide × 0.006/Sample weight in grams) × 100.

#### 2.5.3 Measurement of the thermal stability

Fifteen grams of the sample was weighed in centrifuge tubes and the tubes were placed in an oven at 50°C for 48 h to measure the resistance of becoming two phases due to the heat of the produced samples. The tubes were then centrifuged at 3000 *g* for 10 min. After this step, the oil layer was discarded (Mun et al., 2009). Finally, the stability of the emulsion in terms of percentage was determined using the following equation:

Thermal stability (percentage) = (centrifuged sediment weight/Sample weight) × 100.

#### 2.5.4 Measurement of the physical stability

To measure the physical stability, 10 g of the sample was weighed in centrifuge tubes and the tubes were centrifuged at the room temperature at 5000 *g* for 30 min. After this step, the oil layer was discarded. Emulsion stability in terms of percentage was determined using the following equation (Mun et al., 2009):

Emulsion stability (percentage) = (centrifuged sediment weight/sample weight) × 100.

#### 2.5.5 Determination of the creaming index

To perform this test, immediately after producing the samples, 50 g of mayonnaise from each treatment was poured into McCarthy lid containers and stored at 4°C for 90 days. The creaming index was calculated using the following formula (Konuklar et al., 2004):

Emulsion stability (percentage) = (serum height/total emulsion height) × 100.

#### 2.5.6 Determination of colorimetry

The color characteristics of mayonnaise samples were measured using a colorimeter (X‐RITE) and the L, a, and b parameters were determined, where L indicates the brightness of the sample, which ranges from 0 to −100, b indicates the tendency to yellow (+) and blue (‐), and a indicates the tendency to redness (+) and green (‐) (Ahmad et al., 2009).

#### 2.5.7 Determination of texture properties

The textural characteristics of mayonnaise samples were determined after 1 day. The texture properties of mayonnaise samples were measured using a texture analyzer (TVT 6700, Perten). Samples were carefully filled into acrylic cylindrical containers (inner diameter 50 mm and height 75 mm) to a depth of 50 mm. A cycle of constant speed of one millimeter per second (s) to a depth of 40 mm of the sample was applied and returned to the original state (Worrasinchai et al., 2006). Texture properties, such as stiffness and adhesiveness, were obtained from force–time curve.

#### 2.5.8 Viscosity measurement

The viscosity of the mayonnaise samples was measured using a Brookfield mechanical viscometer (LVDV‐2T) after 1 day. For this purpose, the viscosity of the samples was measured using a T–C spindle from the Helipath Spindle set at a speed of 10 rpm (Ghahremani, 2015).

#### 2.5.9 Determination of sensory evaluation

After the initial tests, 12 semitrained evaluators(10 men and 2 women, aged 23–26 years) were selected. A 5‐point hedonic scale was used for evaluating mayonnaise samples. In this method, each evaluators was given a container containing samples numbered with three‐digit codes, a spoon, a glass of water, a piece of bread along with a scoring form. Each evaluators evaluated all samples randomly and drank water between each sample. Thus, the influential factors of mayonnaise including color (the suitability of the usual mayonnaise color and creaminess), taste, smell, and general acceptance were evaluated (Worrasinchai et al., 2006).

### 2.6 Statistical analysis

All tests were performed with three replications and the data were reported as mean ± *SD*. Statistical analysis was performed using two‐way ANOVA IBM SPSS Statistics 22.0 (IBM SPSS, Inc). The presence of or no significant difference between the values were obtained using Duncan’s test at the level of 0.05.

## 3 RESULTS AND DISCUSSION

### 3.1 Protein levels in different treatments

The initial protein content of clover sprout was equal to 30.81% ± 1.04% and the amount of isolated primary protein of clover sprout was equal to 48.63% ± 1.20%. In addition, the amount of hydrolyzed protein in different treatments (Table 1) was between 50.85% and 91.24%. Based on the results, isolated protein and hydrolyzed protein can be used as value‐added products in food industry or can be used to increase protein levels in animal feed formulas (Aondona et al., 2021). The hydrolyzed sample had higher protein compared to the isolate and clover sprouts seed. The reason for this was protein degradation by hydrolysis followed by centrifugation, led to the separation of nonprotein parts from the hydrolyzed sample (Khantaphant et al., 2011).

### 3.2 Degree of hydrolysis and protein recovery

The results of DH are given in Table 2; the efficiency of enzymatic hydrolysis varies depending on the process conditions, type of enzyme, and time of hydrolysis. As the hydrolysis time increased, the DH increased significantly. Based on the results obtained from the effect of time on protein hydrolysis, with increasing reaction time, enzymatic hydrolysis began in a fast phase and at this stage a large number of peptide bonds were broken. Also, increasing the process time increases the activity of the enzyme and its effect on the substrate (Yu et al., 2018). Since the DH can be used to determine its effect on the functional properties of peptides, it is usually used as an important indicator between different protein hydrolyses. For example, higher DH led to smaller peptides that can have a biological activity such as antioxidant capacity (Wali et al., 2021). The DH by alcalase was higher than flavourzyme. Enzymatic hydrolysis in proteins leads to the formation of peptides with specific functional properties. The side chain effect of their active group R may be related to the greater availability of the group in amino acids. Many different enzymes, such as pepsin, chymotrypsin, alcalase, and flavourzyme, have been used to hydrolyze various plant and animal proteins (Karami & Akbari‐adergani, 2019). The type of enzymes consumed affects the type of peptides produced and their functional properties. The type of enzyme used in the hydrolysis of proteins plays a very important role in the production of bioactive peptides because they directly affect the pattern of protein hydrolysis. The specific activity of enzymes affects the size, amount, and composition of amino acids produced in peptide sequences and it also affects their biological activity (Iravani Mohajeri et al., 2019). PR is one of the important factors in studying the function of enzymes in the hydrolysis of food proteins, which indicates the ability of an enzyme to separate soluble proteins from insoluble types and thus the efficiency of the process during enzymatic hydrolysis is economically important. The results of this study showed that PR (Table 2) by alcalase was higher than flavourzyme. Alcalase enzyme is an alkaline protease enzyme (endoproteinase) that has a microbial origin and therefore shows the appropriate protease properties (Nemati et al., 2012). PR amounts increased significantly with increasing hydrolysis time. The results showed a relationship and correlation coefficient between PR and the DH. This finding indicated that the rate of PR increases with increasing the DH.

**TABLE 2:** Degree of hydrolysis, protein content, and PR of clover sprout protein hydrolysates (CSPH)^a^

Treatment	Degree of hydrolysis (%)	Protein content (%)	Protein recovery (%)
Alcalase 10 min	17.17 ± 0.73^c^	61.21 ± 1.09^c^	12.57 ± 0.22^c^
Alcalase 20 min	26.06 ± 0.46^b^	74.89 ± 0.96^b^	15.38 ± 0.20^b^
Alcalase 30 min	31.90 ± 0.79^a^	91.24 ± 1.30^a^	18.74 ± 0.27^a^
Flavourzyme 10 min	8.24 ± 0.44^e^	50.85 ± 1.23^d^	10.44 ± 0.25^d^
Flavourzyme 20 min	13.23 ± 0.16^d^	63.14 ± 0.91^c^	12.97 ± 0.18^c^
Flavourzyme 30 min	17.98 ± 0.98^c^	76.99 ± 1.05^b^	16.84 ± 0.97^b^

^a,b,c,d:^ Values in same columns with different superscripts are significantly different at *p* < .05.

### 3.3 Amino acid composition

The function of each peptide depends more on its amino acid composition. For example, hydrophobic amino acids (HAAs) have strong antioxidant activity. Particularly due to the breakdown of its imidazole ring, intense radical inhibitory activity is known (Alashi et al., 2014). The total amount of HAAs (Table 3) was 40.97% and 37.60% for alcalase and flavourzyme, respectively. Therefore, it can be argued that proteins hydrolyzed by the alcalase enzyme may have inhibitory effects on several types of free radicals due to higher levels of HAA. In general, differences in raw material, type of enzyme used, and hydrolysis conditions are effective in amino acid composition (Rajabzadeh et al., 2017). Liao et al. (2020) stated that the total HAA of the hydrolyzed protein of the fungus (*Pleurotus geesteranus*) by alcalase and flavourzyme enzymes were 40.66% and 30.96%, respectively.

**TABLE 3:** The amino acid composition of clover sprout protein hydrolysates (g 100 g^‐1^; 30 min)

Amino acid(g 100 g^−1^)	Alcalase	Flavourzyme	FAO/WHO, 1990
Histidine ^a^	2.25	2.95	
Isoleucine ^a^	3.97	3.15	2.8
Leucine^a^	7.78	7.12	6.6
Lysine^a^	5.96	6.82	5.8
Methionine^a^	0.45	0.32	
Phenyl alanine^a^	5.45	4.55	6.3
Tyrosine	3.97	3.4	1.1
Threonine^a^	6.55	7.35	3.4
Arginine^a^	4.59	5.45	
Valine^a^	5.52	5.09	3.5
Aspartic acid	18.99	17.58	
Glycine	4.68	5.13	
Proline	7.95	8.58	
Serine	4.99	5.78	
Alanine	5.85	5.39	
Cystein	0.35	0.59	
Glutamic acid	10.59	9.95	
Total amino acid	99.92	99.20	
Essential amino acid/nonessential amino acid	0.74	0.75	
Essential amino acid/total amino acid	42.55	43.15	
HAA^b^	40.97	37.60	

^a^ Essential amino acids

^b^ Total hydrophobic amino acids (alanine, valine, isoleucine, leucine, tyrosine, phenylalanine, proline, methionine, and cysteine).

In total, the highest levels of nonessential amino acids for alcalase and flavourzyme were aspartic acid 18.99% and 17.58%, respectively, and the highest levels of essential amino acids for these enzymes were leucine 7.78% and 7.12%, respectively. Purwin et al. (2015) stated that the highest levels of essential amino acids for red clover protein were leucine (5.35%) and the highest levels of nonessential amino acids were aspartic acid (12.08%). Penkov et al. (2003) stated that the highest levels of essential amino acids for the protein of different clover species were leucine (between 6.89% and 7.78%), and the highest levels of essential amino acids were aspartic acid (between 13.23% and 19.45%). According to studies, amino acid levels are different for clover species, but in all of these species, the highest amino acid levels were related to aspartic acid.

The total amount of amino acids (Table 3) was 99.92 and 99.20 for alcalase and flavourzyme, respectively. According to FAO/WHO/UNU (2007), the ratio of essential amino acid to total amino acids should not be less than 40% and also the amount of essential to nonessential amino acids should not be less than 0.6. According to the results, the hydrolyzed protein has a suitable amino acid composition. The ratio of essential to nonessential amino acids of alcalase and flavourzyme is 0.74 and 0.75, respectively, and the ratio of essential amino acid to the total available amino acid is 42.55 and 43.14, respectively. Also, the levels of threonine, valine, isoleucine, leucine, tyrosine, histidine, and lysine in both hydrolyzed proteins were higher than the FAO/WHO/UNU (2007) recommendations for animal proteins. It was limited only in relation to phenylalanine. All these results indicate that clover sprout protein has a high nutritional quality and may be used as a source of protein in human and animal diets.

### 3.4 Approximate analysis of mayonnaise

Although the amount of moisture (Table 4) in different treatments was not significantly different from each other, the amount of fat and ash decreased when replacing eggs with clover protein (control treatment 67.55%), and the lowest amount of fat was observed in the treatment containing 9% protein (63.58%). Protein levels increased in the control treatment were lower than other treatments (2.02%) and the highest protein levels were observed in the treatment containing 9% protein (5.27%). Eggs contain approximately 70% water, 12.5% protein, 12% fat, and 11.5% ash (Pradhananga & Adhikari, 2015). In this study, clover protein was first dissolved in water so that it has 70% moisture like eggs, then it was replaced with eggs. Therefore, hydrolyzed clover protein contained approximately 70% water, 27.32% protein, 0.16% fat, and 1.95% ash. Therefore, it is necessary to increase the amount of protein and decrease the amount of fat and ash, and keep the amount of moisture constant. Previously, El‐Bostany et al. (2011) reported a reduction in total fat content in emulsion‐based products when the fat components were replaced with nonfat ingredients. Similar results were observed by Unnikrishnan et al. (2020). They also reported that by replacing eggs with the hydrolyzed protein from yellowfin tuna meat in mayonnaise increased the protein content from 3.58% to 6.16% and decreased the fat content from 50.22% to 45.29%.

**TABLE 4:** Proximate composition in different treatment of mayonnaise^a^

Treatment	Moisture (%)	Protein (%)	Fat (%)	Ash (%)	Carbohydrate (%)
Control	24.17 ± 0.73^a^	2.02 ± 0.07^d^	67.55 ± 0.86^a^	0.82 ± 0.03^a^	5.42 ± 0.04^c^
CSPH 1%	23.89 ± 1.80^a^	3.6 ± 0.13^c^	65.87 ± 0.54^b^	0.69 ± 0.04^b^	5.95 ± 0.09^b^
CSPH 2%	24.38 ± 0.61^a^	4.3 ± 0.21^b^	64.88 ± 0.71^b^	0.64 ± 0.01^b^	5.79 ± 0.08^b^
CSPH 3%	24.19 ± 1.21^a^	5.27 ± 0.17^a^	63.58 ± 0.38^c^	0.63 ± 0.02^b^	6.31 ± 0.08^a^

^a,b,c,d:^ Values in same columns with different superscripts are significantly different at *p* < .05.

### 3.5 Color index of mayonnaise

**FIGURE 1:**
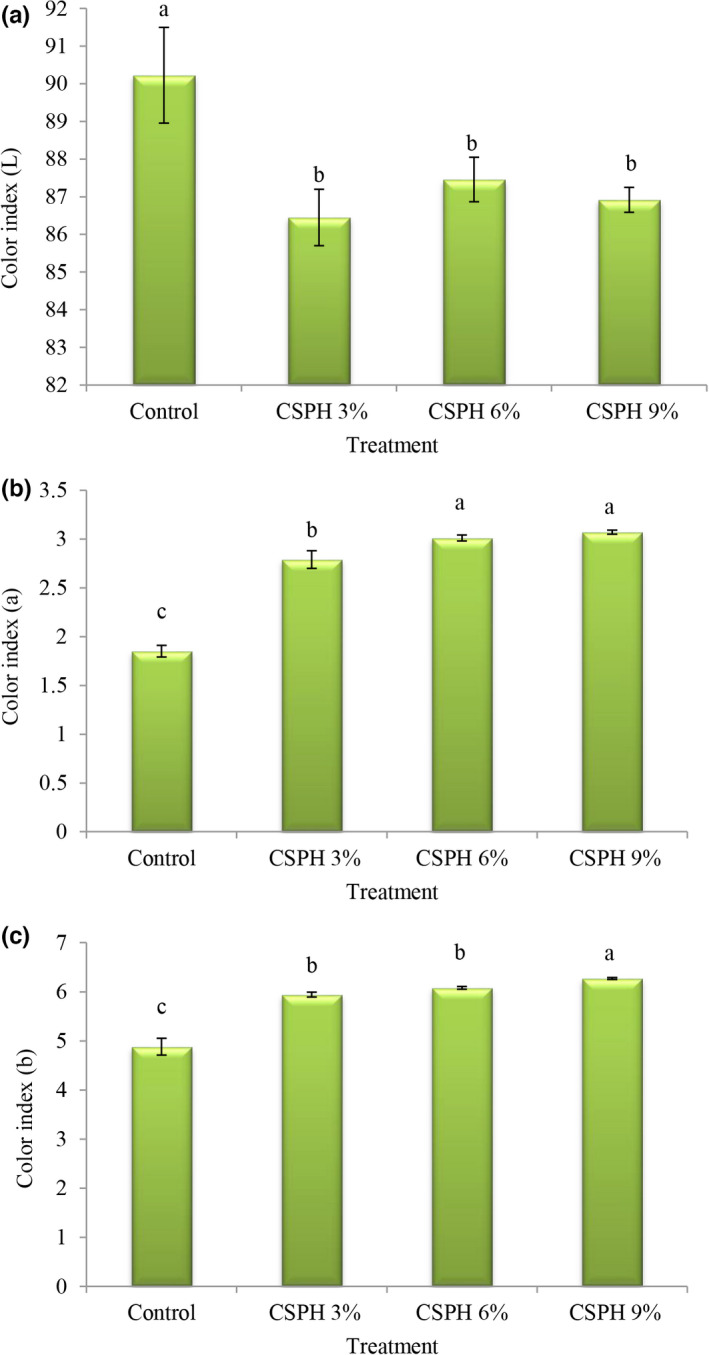
Comparison of average of mayonnaise treatments in terms of color index L (a), a (b), and b (c)

The color index L* (Figure 1a) indicates the brightness (black to white) of the product. The results related to the values of the L in the control treatment were higher than in other treatments (90.23); by replacing eggs with clover protein, the values of the color index (L*) decreased. The color index a* (Figure 1b) indicates the color change from green to red of the product. The results related to the values of the color index (a*) in the control treatment were less than in other treatments (1.85; *p* < .05); by increasing the protein concentration, the values of the color index (a*) increased and the highest values of the color index (a*) were observed in treatments containing 6% and 9% protein. The color index b* (Figure 1c) indicates the of color change from blue to yellow. Also, the results indicated that the values of the color index (b*) in the control treatment was significantly less than in other treatments (4.88); by increasing the protein concentration, the values of the color index (b*) decreased and the lowest value of the color index (b*) was observed in the treatment containing 9% protein (6.27).

In general, the replacement of eggs with the hydrolyzed protein reduced the brightness and increased redness and yellowness of mayonnaise samples, and the color of the product with a higher level of substitution resulted in a reddish‐brown color in the mayonnaise. The change in the color index after the replacement with hydrolyzed clover protein is due to the yellowish‐brown color of the protein. Typically, when a product deviates from the standard color range, the attractiveness of the product is affected and this variation also affects the degree of acceptance of protein‐containing samples by the consumer. Similar results were reported by Unnikrishnan et al. (2020). They also reported that by replacing eggs with the hydrolyzed protein from yellowfin tuna meat in mayonnaise, the color index (L*) values decreased but (a*) and (b*) increased.

### 3.6 Texture characteristics of mayonnaise

**FIGURE 2:**
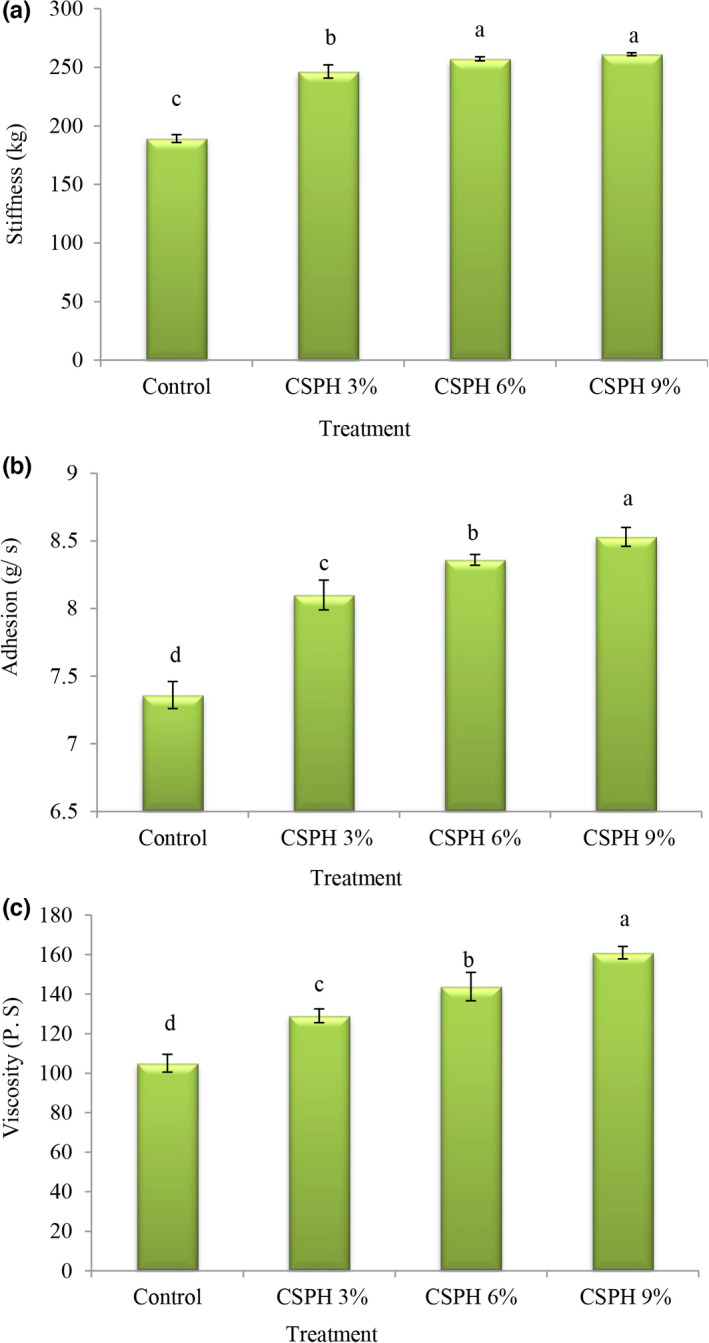
Comparison of mayonnaise treatments averages in terms of stiffness (a), adhesion (b), and viscosity (c)

Based on statistical analysis, the amount of stiffness (Figure 2a) in the control treatment was less than in other treatments (189.24); with increase in the protein concentration, the amount of stiffness increased and the highest values of stiffness were observed in the treatment containing 6% and 9% clover protein. Also, the values of adhesiveness (Figure 2b) in the control treatment were lower than in other treatments (7.36 g/s) and the highest values of adhesiveness were observed in the treatment containing 9% protein (8.53 g/s; *p* < .05). According to the obtained results, it can be stated that by increasing clover protein, mayonnaise had with more firmness and cohesiveness compared with the sample containing eggs (control). These results are probably due to the increased viscosity of samples containing high levels of clover protein. The addition of protein as an egg substitute significantly increases the viscosity and forms a stable oil‐in‐water emulsion; therefore, the oil droplets are preserved and their coagulation is reduced compared to the sample containing eggs (Nikzade et al., 2012). Previously, Rahbari et al. (2013) stated that mayonnaise containing wheat germ protein isolate has higher stiffness and adhesiveness than the in control sample.

### 3.7 Viscosity of mayonnaise

Based on statistical analysis, the viscosity values (Figure 2c) in the control treatment were lower than in other treatments (104.99 Pa. s), and with the replacement of eggs with clover protein, the viscosity values increased and the highest viscosity values were observed in the treatment contained 9% protein. (160.99 Pa. s). The higher viscosity observed in the sample containing clover protein may be due to the higher total protein content as well as the higher ability of water to bind to it, thus preventing its continuous phase mobility. Gaonkar et al. (2010) observed higher viscosity for mayonnaise samples prepared using whey protein concentrate and whey protein isolate compared to the control. Similar results were reported by Unnikrishnan et al. (2020). They also reported that viscosity increased by replacing eggs with the hydrolyzed protein from yellowfin tuna meat in mayonnaise.

### 3.8 Acidity and pH of mayonnaise

**FIGURE 3:**
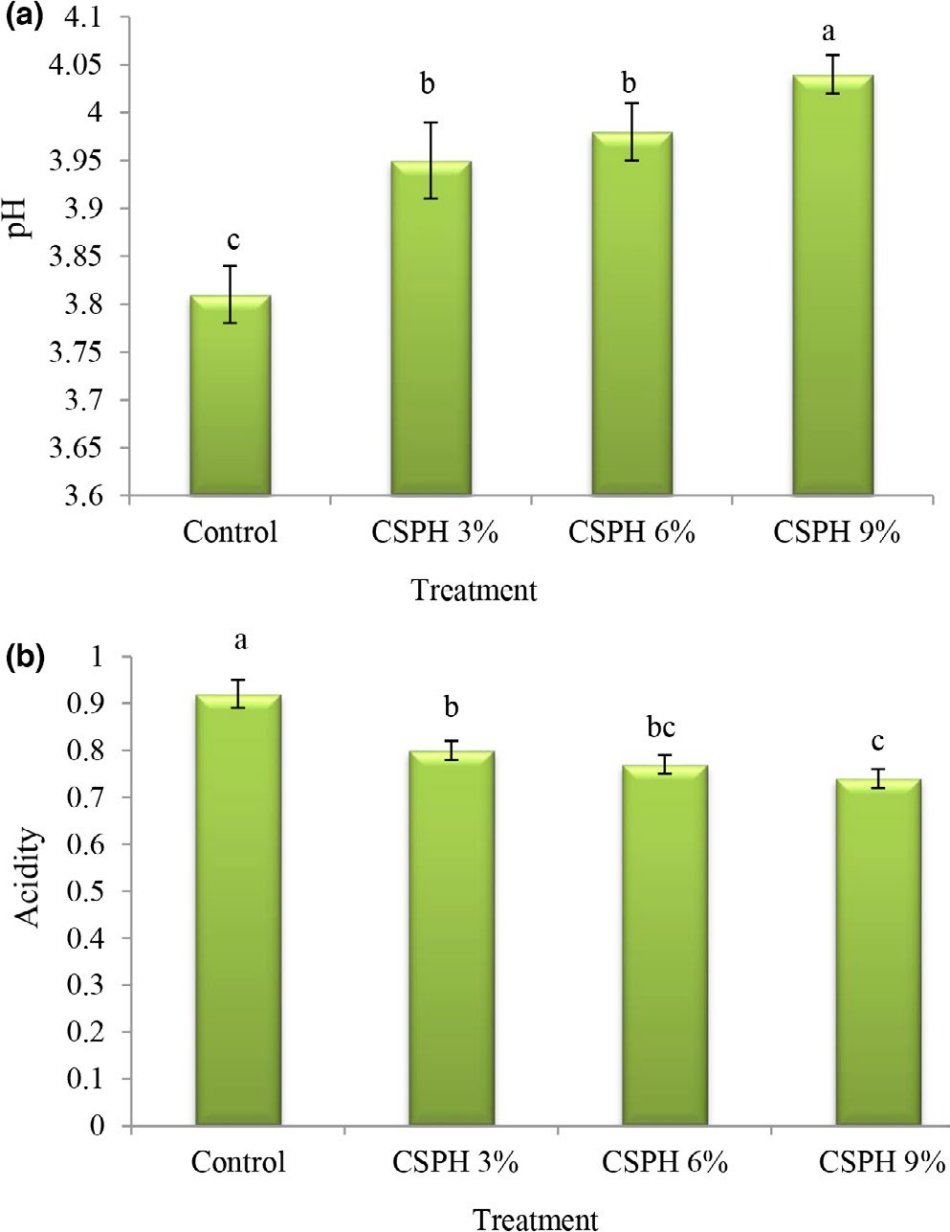
Comparison of mayonnaise treatments averages in terms of pH (a) and acidity (b)

Acidity and pH are very important chemical factors in salad dressings, including mayonnaise, for which a certain range is defined in the Iranian National Standard. According to the Iranian National Standard (No. 2454), the pH of mayonnaise should not be more than 4.1 and the total acidity should not be less than 0.6 in terms of grams per hundred grams of acetic acid (ISIRI, 2014) because raising the pH may allow the growth of pathogenic bacteria, and if the acidity is more than 1.5%, the resulting mayonnaise will have an unpleasant taste. The optimum acidity is 0.7%–1.2%. The values of pH (Figure 3a) in the control treatment were lower than in other treatments (3.81), and with the replacement of eggs with clover protein, pH values increased and the highest pH values were observed in the treatment containing 9% protein (4.04). Also, the acidity values (Figure 3b) in the control treatment were higher than in other treatments (0.92), and by replacing eggs with clover protein, the acidity values decreased and the lowest acidity values were observed in the treatment containing 9% protein (0.74). In general, all treatments had a standard range. The higher pH values after replacing eggs with the protein are probably due to the effect of protein pH on the total pH of the product. When preparing protein hydrolysate, the pH of the protein deposition was brought to a neutral range before drying in a freeze dryer, but the pH of the egg is about 6.24 (Unnikrishnan et al., 2020). Therefore, increasing the pH and decreasing the acidity is obvious.

### 3.9 Stability of mayonnaise

**FIGURE 4:**
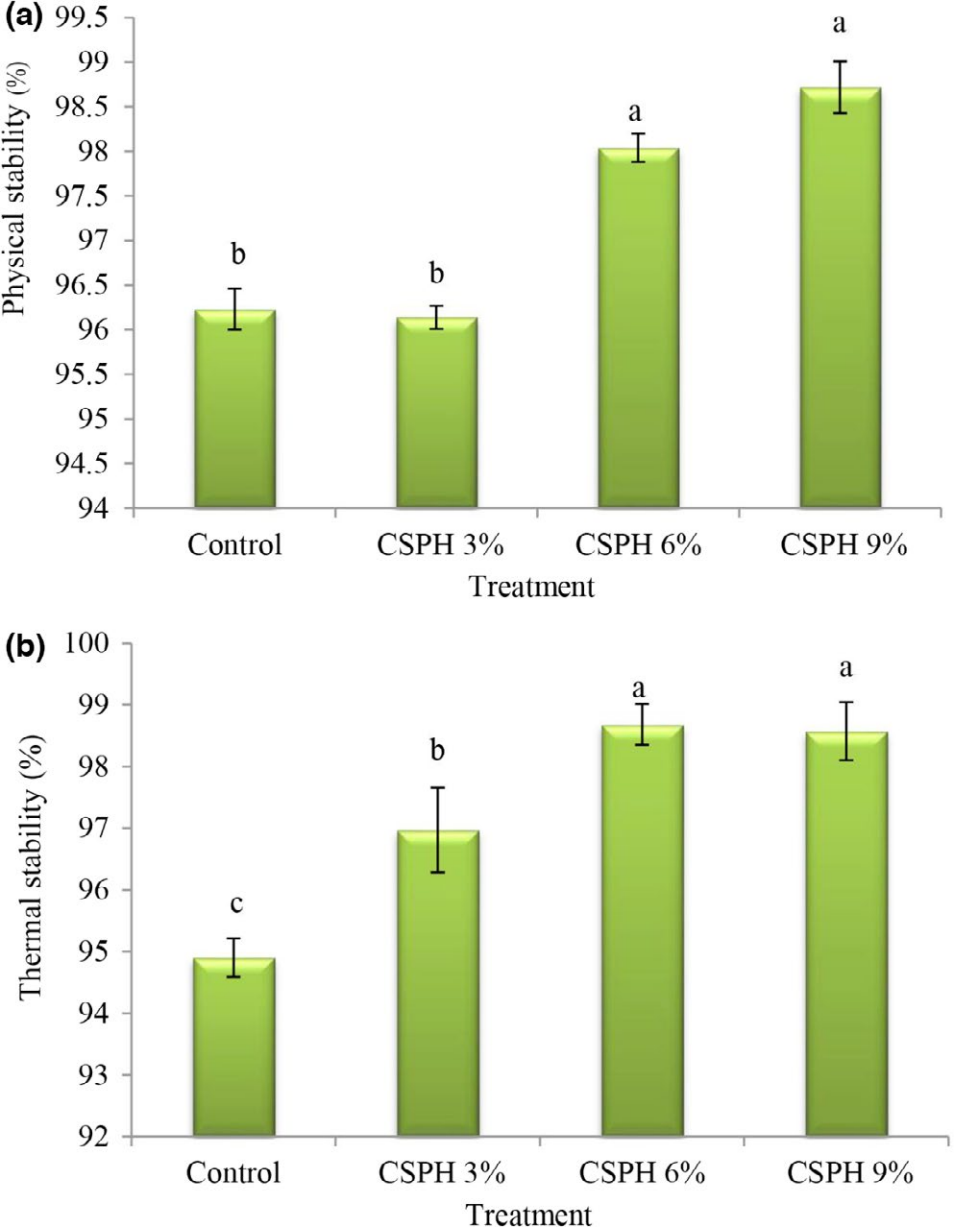
Comparison of mayonnaise treatments averages in terms of physical stability (a) and thermal stability (b)

Stable emulsion refers to an emulsion in which coalescence and flocculation do not occur. The physical stability (Figure 4a) in control and treatment containing 3% protein was less than other treatments, and by replacing eggs with clover protein at concentrations of 6% and 9%, physical stability values increased. Also, the values of thermal stability (Figure 4b) in the control treatment were significantly lower than in other treatments, and by replacing eggs with clover protein, the values of thermal stability decreased and the highest values of thermal stability were observed in the treatment containing 6 and 9% protein. These findings are consistent with the results of Herald et al. (2009) who observed that the stability of mayonnaise samples at 50% replacement of eggs with isolated protein of wheat and whey protein was significantly lower than the stability of 100% replacement samples. This may be due to the increase in continuous phase viscosity following the addition of clover protein, which prevents emulsion fusion and instability by reducing the droplets of oil droplets and increases stability (Herald et al., 2009). The instability of the mayonnaise emulsion is mostly due to the merging of the emulsion droplets with each other and the increase in particle diameter. As a result, reducing the ratio of surface to volume reduces friction among the emulsion droplets and leads to emulsion instability. The most important factor in preventing coagulation is the presence of sufficient and strong repulsive forces among oil droplets by emulsifying agents such as proteins and polysaccharides so that increasing viscosity reduces the movement of oil droplets and ultimately increase stability. Unnikrishnan et al. (2020) also reported that replacing eggs with the hydrolyzed protein from yellowfin tuna meat in mayonnaise increased mayonnaise stability, and Rahbari et al. (2013) reported that mayonnaise containing wheat germ protein has higher physical and thermal stability than in the control treatment.

### 3.10 Sensory evaluation of mayonnaise

**FIGURE 5:**
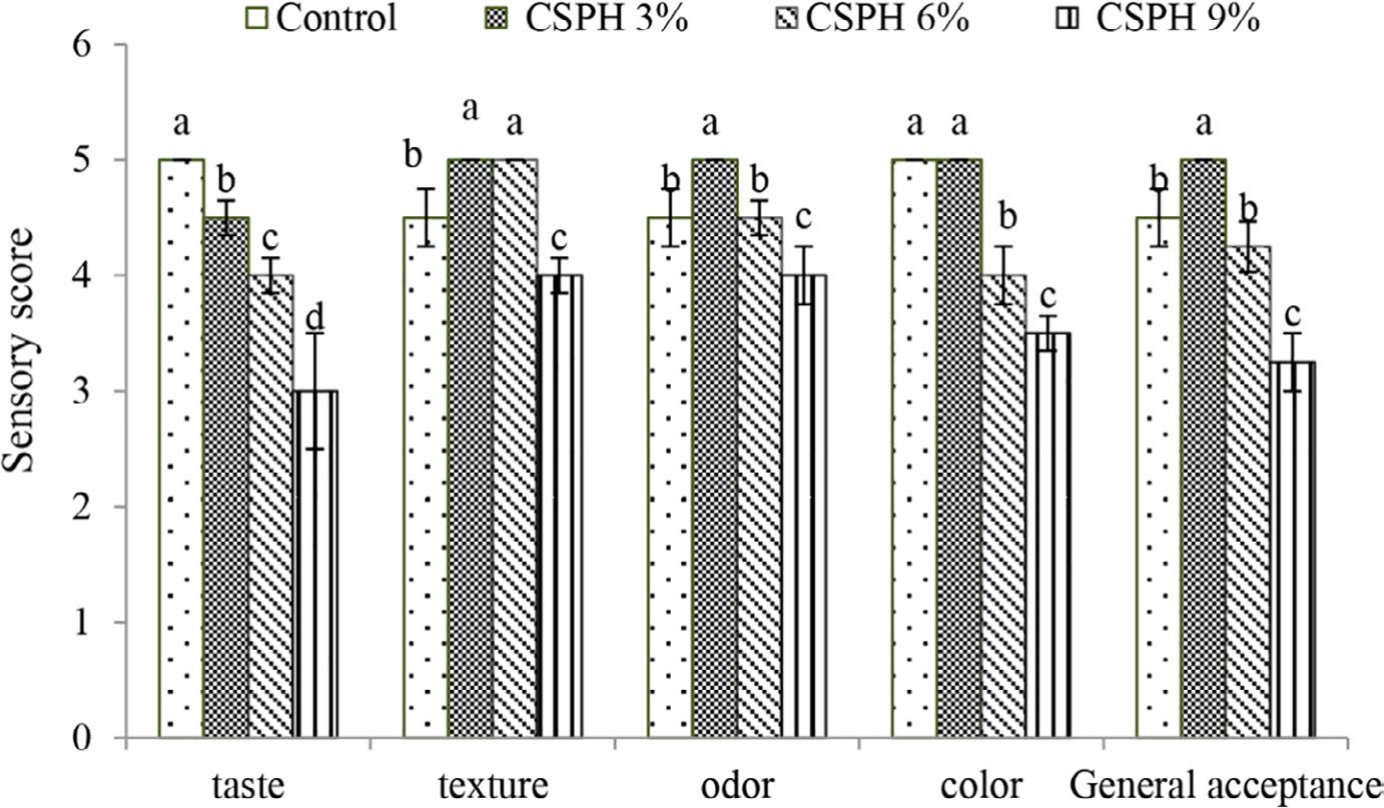
Comparison of mayonnaise treatments averages in terms of sensory score

Sensory acceptance is one of the most important factors for ready‐to‐eat products such as mayonnaise. The most important sensory parameter of a food emulsion is its overall appearance, which is a collective effect of color as well as its texture. According to the results of replacing eggs with the hydrolyzed protein in mayonnaise at the level of 3%, it improved the sensory properties (Figure 5; except for taste), but with increasing concentration, the sensory score decreased, so that the lowest sensory score values were found at 9% protein and this treatment was not approved by the evaluators, So the best sensory score including color, taste, smell, and general acceptance were observed in the treatment with 3% clover protein, the lower sensory score was observed in treatment containing 3% sprout protein. Similar results related to the replacement of eggs with the hydrolyzed protein from yellowfin tuna meat in mayonnaise were reported by Unnikrishnan et al. (2020). Challenges associated with the development of emulsions replacing traditional products with comparable sensory attributes like appearance, texture, and flavor have been previously reported by several authors (Puligundla et al., 2015; Mozafari et al., 2017).

## 4 CONCLUSION

The results showed that by replacing eggs with hydrolyzed clover protein increased the protein content, pH, physical stability, thermal stability, viscosity, stiffness and adhesiveness, redness and yellowness index, and the decreased fat content, brightness, and acidity. In total, treatment 3: 3% egg + hydrolyzed clover protein 6% and treatment 4: 0% egg + hydrolyzed clover protein 9% had better properties; however only treatment 3 was approved by the evaluators. The results indicate that using clover protein up to 6% level can satisfactorily remove most of the mayonnaise egg and produce low‐egg mayonnaise. The positive results of this study are by employing suitable hydrolyzed clover protein instead of egg, the cholesterol of mayonnaise decreased and its nutritional value increased, but had a negative effect on some properties when compared with the main formulation.

## CONFLICT OF INTEREST

The authors declare that they do not have any conflict of interest.

## STUDIES INVOLVING HUMAN AND ANIMAL SUBJECTS

Human and animal testing is unnecessary in this study.

## INFORMED CONSENT

Written informed consent was obtained from all participants.

## Data Availability

All the data used in this study can be made available upon reasonable request.
